# Association and Gene–Gene Interactions Study of Late-Onset Alzheimer’s Disease in the Russian Population

**DOI:** 10.3390/genes12101647

**Published:** 2021-10-19

**Authors:** Anna Bocharova, Kseniya Vagaitseva, Andrey Marusin, Natalia Zhukova, Irina Zhukova, Larisa Minaycheva, Oksana Makeeva, Vadim Stepanov

**Affiliations:** 1 Tomsk National Research Medical Center, Research Institute of Medical Genetics, 634050 Tomsk, Russia; vagaitzeva.xenia@yandex.ru (K.V.); andrey.marusin@medgenetics.ru (A.M.); larisa.minaycheva@medgenetics.ru (L.M.); vadim.stepanov@medgenetics.ru (V.S.); 2 Department of Neurology and Neurosurgery, Faculty of General Medicine, Siberian State Medical University, 634050 Tomsk, Russia; znatali@yandex.ru (N.Z.); irzhukova@inbox.ru (I.Z.); 3 Nebbiolo Center for Clinical Trials, 634009 Tomsk, Russia; oksana.makeeva@nebbiolomed.ru

**Keywords:** Alzheimer’s disease, APOE, case–control study, GWAS, SNP

## Abstract

Alzheimer’s disease (AD) is a neurodegenerative disorder, and represents the most common cause of dementia. In this study, we performed several different analyses to detect loci involved in development of the late onset AD in the Russian population. DNA samples from 472 unrelated subjects were genotyped for 63 SNPs using iPLEX Assay and real-time PCR. We identified five genetic loci that were significantly associated with LOAD risk for the Russian population (*TOMM40* rs2075650, *APOE* rs429358 and rs769449, *NECTIN* rs6857, *APOE* ε4). The results of the analysis based on comparison of the haplotype frequencies showed two risk haplotypes and one protective haplotype. The GMDR analysis demonstrated three significant models as a result: a one-factor, a two-factor and a three-factor model. A protein–protein interaction network with three subnetworks was formed for the 24 proteins. Eight proteins with a large number of interactions are identified: APOE, SORL1, APOC1, CD33, CLU, TOMM40, CNTNAP2 and CACNA1C. The present study confirms the importance of the APOE-TOMM40 locus as the main risk locus of development and progress of LOAD in the Russian population. Association analysis and bioinformatics approaches detected interactions both at the association level of single SNPs and at the level of genes and proteins.

## 1. Introduction

Alzheimer’s disease (AD) is a neurodegenerative disorder and the most common cause of dementia. The disease is characterized by the loss of neurons and synaptic connections in the cortex and certain subcortical areas. AD is clinically characterized by decline in progressive executive function, perceptual speed, short-term memory and other cognitive capacities [1]. Degradation of cognitive functions has a major impact on an individual’s quality of life. According to the World Alzheimer Report 2018, there are over 50 million people suffering from AD or other dementias in the world [2]. Each year, approximately 5–7 million new cases of AD are registered in the geriatric population [3].

Two forms of AD are traditionally distinguished: early onset (EOAD, onset <65 years old) and late-onset (LOAD, onset >65 years old). EOAD accounts for 1–6% of AD cases. These cases are familial and are associated with classical Mendelian patterns of inheritance. The amyloid precursor protein (*APP*), presenilin1 (*PSEN1*), and presenilin2 (*PSEN2*) genes are primarily implicated in EOAD. As for the later onset form of the disease, both genetic and environmental factors are considered to impact the disease risk profile. LOAD constitutes approximately 95% of AD cases [4]. Heritability for LOAD is estimated to be between 58% and 79%, on the basis of a twin study design [5]. The genetic complexity of LOAD should not be underestimated. Due to general efforts, it has become clear that pathogenesis of LOAD is multi-faceted and is not limited to one set of simple molecular interactions.

Common neurological and psychiatric disorders, including AD, schizophrenia, bipolar disorder, Parkinson’s disease are now the subject of intensive studies. Over the last few decades great progress in researching of susceptibility genetic loci to common mental and neurological diseases has been made using new technologies and methods: genome-wide association studies (GWAS), large meta-analysis, whole-exome/genome sequencing (GWAS, meta-analysis, NGS). 

Alzheimer’s disease and schizophrenia are two common diseases of the brain with significant differences in neuropathology, etiology and symptoms. Data on the common links in the pathogenesis of AD and schizophrenia are accumulating [6]. Thus, statistical evidence has shown that AD and schizophrenia have a specific molecular background. Transcriptome studies have found a similar pattern of gene expression in the superior temporal gyrus in AD and schizophrenia [7]. Recently, GWASs have identified many genomic loci characterized by small effect sizes associated with LOAD: *CLU, CR1, PICALM, BIN1, ABCA7, CD33* and others [8,9,10]. It is important to keep in mind that genetic association studies have often produced false findings, and thus need validation in multiple sample sets. On the other hand, it is known that most GWAS SNPs in the associated loci with disease cannot be explained on the basis of known pathological mechanisms. Next-generation sequencing has assisted the identification of rare susceptibility modifying alleles in APP, TREM2, and PLD3 [11]. However missing heritability for LOAD remains extensive. While great progress has been made with respect to understanding the genetic landscape of LOAD, the connection between the genes and variants promoting to the risk of the disease remains unclear. The data accumulation and the use of new bioinformatics approaches including those implemented in publicly available resources can help with this problem. The biological roles in metabolic pathways of all newly identified variants, as well as functionally related genes, can be assessed using ALIGATOR, KEGG, GO, STRING, GeneMANIA [12,13,14,15,16]. Exploring potentially new protein interactions in common mental and neurological diseases using bioinformatic strategies presents potential interest. Genetic risk factors could be analyzed and integrated in terms of biological pathways and functions to better understand the contribution of genetics to the general background of neurological and mental diseases. Protein interaction data, derived from the GWAS database, can be used to build protein–protein interaction (PPI) networks from genes associated with AD, schizophrenia and cognitive performance. PPI network analysis could be an effective and powerful approach to identify potential biological pathways or key genes involved in the link of neurological and mental diseases.

In addition to the complexities of LOAD associated with numerous interconnected environmental and genetic factors, the genetic architecture of LOAD differs between ethnic groups. Our prior study estimated genetic diversity of sixteen native populations of North Eurasia using a panel of genetic markers significantly associated with AD and Schizophrenia [17].

Here we follow up on our prior research in a new cohort of the AD group and controls. In the present study, we aimed to analyze 63 of the previously reported statistically significant SNP markers associated with AD in approximately 450 Russian samples from well-characterized LOAD case–control set. In addition, our focus was on the identification of the molecular and functional pathways of these SNP markers using various statistical and bioinformatics resources.

## 2. Materials and Methods

### 2.1. Samples

This study involved 185 unrelated LOAD patients (mean age 72.15 ± 7.87 years) recruited and evaluated at the Department of Neurology and Neurosurgery (Siberian State Medical University, Tomsk, Russia). Subjects were included in the study after a diagnosis of LOAD according to the International Classification of Diseases, Tenth Revision, Clinical Modification (ICD-10-CM), the diagnostic and statistical manual of mental disorders, fourth edition, text revision (DSM-IV TR) [18] and the NINCDS-ADRDA Alzheimer’s Criteria (the National Institute of Neurological and Communicative Diseases and Stroke and the Alzheimer’s Disease and Related Disorders Association) [19]. Structural imaging based on magnetic resonance is a part of the clinical assessment of patients with suspected AD. The presence of focal symmetrical medial temporal atrophy has predictive value for AD. Our study included the cases with MRI images showing the characteristic atrophy of the hippocampus. Cases had a minimum age at disease onset of 65 years. Control group consisted of 287 unrelated cognitively normal elderly Russian participants (mean age 71.8 ± 5.7 years). The same procedures for the control group were performed as for the case group to exclude the LOAD diagnosis. Accordingly, history, neurologic examinations, magnetic resonance images of brain volumes were examined for the control group. Additionally, the MoCA test was conducted for the control group to exclude mild cognitive impairment. The MoCA was developed to be more sensitive to mild cognitive impairment in geriatric populations than other screeners (like the Mini Mental State Examination-MMSE). MoCA scores ranged between 0 and 30 points, and the higher scores indicated the better cognitive performance. In our study, the control group included people with the MoCA score ≥ 23. Controls were recruited and evaluated at the Nebbiolo Center for Clinical Trials (Tomsk).

All of the studied individuals, cases and controls were of the same ethnic (Russians) and geographical origins, living in the Tomsk region of the Russian Federation. Table 1 presents the main demographic parameters of the studied groups. The study protocol was approved by the Scientific Ethics Committee of the Research Institute of Medical Genetics (Tomsk National Research Medical Center of the Russian Academy of Sciences) (protocol number 2017/108). Individuals or legal guardians signed a written informed consent form after the study objectives and procedures had been explained.

### 2.2. Genotyping

Genomic DNA was extracted from the peripheral venous blood using phenol–chloroform extraction method. For this study, two multiplex panels of SNP markers that showed repeated association with cognitive performance, AD or/and schizophrenia in the GWAS were formed (see Table 2). Genetic markers were selected based on certain and clear criteria. First of all, we selected loci reaching a genomic value (*p* < 5.00 × 10^−7^) for the phenotype (cognitive functions, AD and/or schizophrenia in GWAS). The next requirement concerned the type of marker: only SNPs were selected. The minor allele frequency (MAF) should have been ≥ than 5% in at least one population group of the HapMap project or the 1000 Genomes project. Selected markers should form multiplexes using the Assay Design Suite v2.0 genotyping tool (https://agenacx.com/online-tools/, accessed on 6 August 2021). All patients and controls were genotyped for 62 SNPs using iPLEX Assay following the recommended protocol by the manufacturer (Agena Bioscience™, San Diego, CA, USA). In addition to 62 SNPs, genotyped by MALDI/TOF mass spectrometry, rs7412 of APOE was genotyped by real-time PCR using the TaqMan^®^ SNP Genotyping Assay (Applied Biosystems, Foster City, CA, USA).

### 2.3. Statistical Analyses

Two SNPs (SORL1 rs11218343, TCF4 rs1261117) were excluded from the final analysis because they showed genotype call rates < 87%. Per-marker genotype call rates were higher than 95% for all the rest of the markers. The genotype distributions of 3 loci (*LUZP2* rs1021261, *CNTNAP2* rs10273775, *GRIN2B* rs2160519) were not consistent with the Hardy–Weinberg equilibrium in the control group. Therefore, these three polymorphisms were excluded from further analyses.

Pearson’s chi-squared test was performed to verify the Hardy–Weinberg equilibrium of each SNP in LOAD patients and controls. The genetic variants in the control group which were not in the Hardy–Weinberg equilibrium (*p* < 0.05) were excluded from the analysis. Allele and genotype frequencies of each SNP were compared between LOAD cases and controls using the chi-squared (χ^2^) test. The odds ratios (OR) and 95% confidence intervals (CIs) were calculated to assess the relative risk. The Benjamini and Hochberg false discovery rate method was used for multiple testing corrections [20]. A *p* value threshold of 0.05 was used to determine significance.

**Figure 1:**
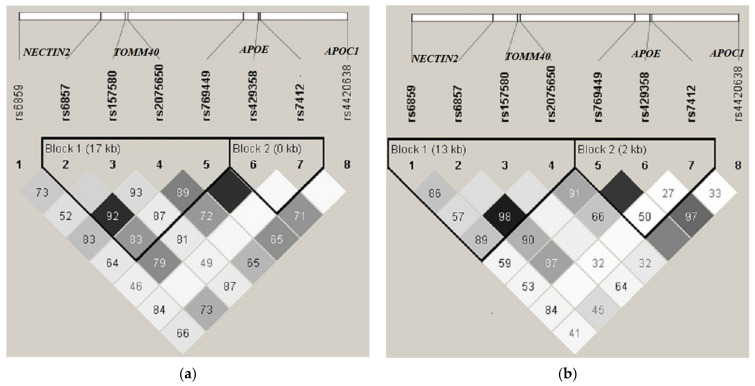
The structure of linkage disequilibrium in the genomic region of the *NECTIN2-TOMM40-APOE* locus: (**a**) tThe structure of LD for the cases; (**b**) the structure of LD for the controls. Linkage disequilibrium was measured by the D’ statistic: black—a strong link (D’ = 1, LOD > 2), grey—a significant link (D’ < 1, LOD > 2), white—poor link (D’ < 1, LOD < 2). A D’ value of 100 indicates a complete LD between 2 markers.

The linkage disequilibrium (LD) between SNP pairs in the genomic region of APOE- TOMM40 locus was quantified using Haploview version 4.2 software [21]. Haplotype frequencies were determined using the EM algorithm. The LD block structure was determined using the Solid Spine method provided by the Haploview 4.2. The degree of genetic linkage between the 8 SNPs in study groups was estimated as Lewontin’s coefficient D’ and Pearson’s correlation coefficient r^2^, where no color (D’ = 0) indicates that LD is weak or nonexistent and the black (D’ = 1) indicates that there exists strong pairwise LD between SNPs (Figure 1).

Generalized multifactor dimensionality reduction (GMDR) analysis was carried out for the detection and characterization of gene–gene interactions. This is a generalized MDR framework based on the score of a generalized linear model [22]. The best interaction model was selected on the basis of maximum testing balanced accuracy (TBA) and cross-validation consistency (CVC). Permutation (p) results were considered to be statistically significant at the 0.05 level. In our study, we used open-source software GMDR (http://sourceforge.net/projects/gmdr, accessed on 5 August 2021).

The protein–protein interaction network of the studied proteins was constructed with the online analysis tool STRING v11.0 (http://www.string-db.org/, accessed on 5 August 2021). The interactions include indirect (functional) and direct (physical) associations, which are derived from four sources: experiments, genomic context, co-expression (conserved), and previous knowledge. Biological processes, cellular components and molecular functions were analyzed by using a freely available system PANTHER (Released 2020-12-18, http://www.pantherdb.org/, accessed on 5 August 2021). STRING and GO analyses included 41 genes. SNPs of these genes were genotyped in the present study (Table 2). The analyses did not include markers from intergenic regions.

## 3. Results

The minor allele frequencies of 58 SNPs in LOAD patients and in controls are presented in a Table 2. Almost all the allele and genotype frequencies for the investigated loci were within the range reported in other populations of the European descent in the 1000 Genomes project (https://www.ncbi.nlm.nih.gov/variation/tools/1000genomes/, accessed on 5 August 2021), with the exception of three genetic variants. The G allele of rs2075650 at *TOMM40* gene has higher frequency (28%) in the Russian population in comparison with other populations of the European origin in the 1000 Genomes project (16%). The minor allele C of rs7984606 at *KLHL1* gene has lower frequency (0.4%) in the Russian population than other populations of European descent in the 1000 Genomes project (1%). The allele A of *DCHS2* rs1466662 has lower frequency (35%) in the Russian population than other populations of European origin in the 1000 Genomes project (69%).

The statistical power of the study was estimated for the Genetic Association Study (GAS) Power Calculator (http://csg.sph.umich.edu/abecasis/gas_power_calculator/, accessed on 5 August 2021) assuming an odds ratio (Genotype Relative Risk) of 1.5, allele frequencies 18% in AD cases, and a significance level of 0.05. The sample size of this study was estimated to have 80% power to detect a positive association for 36 variants with 18% frequency in AD cases. Power to detect an odds ratio of 1.5 or greater was 80% for six of the nine SNPs in Table 2, showing non-significant results after correction for multiple testing (FDR) for three studied variants: *FBXO40* rs3772130, *CR1* rs6656401, *NECTIN2* rs6859. The reporting of variants with low frequency which are not sufficiently powered is justified not only by detecting associations with the disease but by estimating allele frequencies of these markers in cases and controls belonging to specific population.

Out of the 58 markers tested, nine showed significant association to LOAD (Table 3). Two variants demonstrated marginal *p* values for the allele and/or genotype frequency difference between the LOAD and control subjects: *FBXO40* rs3772130 and *NECTIN2* rs6859. After correction for multiple testing the results remained statistically significant for five genetic variants in three genes: *TOMM40* rs2075650, *APOE* ε4, rs429358 and rs769449, *NECTIN* rs6857 (Table 3). All of these markers were located in the same region of the chromosome 19.

Due to the fact that it is important for the LD analysis that the markers are in the same chromosome region, we included five SNPs in the LD analysis, which in our study were significantly associated with LOAD: *TOMM40* rs2075650, *APOE* rs7412, rs429358 and rs769449, *NECTIN* rs6857. All of these markers were located in the same region of chromosome 19 (the *APOE-TOMM40* region). Markers *TOMM40* rs157580, *NECTIN* rs6859, APOC1 rs4420638 were genotyped, but were not significantly associated with the LOAD in our study. These markers were also analyzed using the LD analysis, as they were also located in the *APOE-TOMM40* region of chromosome 19, like the other 5 markers. The theoretically possible number of haplotypes was 256. A total of 44 haplotypes were detected in our samples. Twenty haplotypes were revealed in the control group, while 24 haplotypes were found in the cases. Fourteen identical haplotypes were observed in both groups. Identical haplotypes and their frequencies are presented in a Table 4.

**Figure 2:**
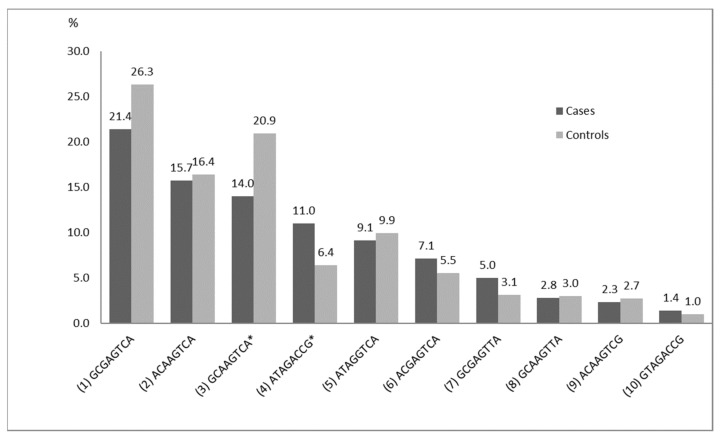
Haplotype frequencies for the LOAD patients and the control group. * Statistically significant differences between the cases and the controls (*p* < 0.05).

Figure 2 demonstrates haplotypes with frequencies above 1% in the case and the control groups.

The number of main haplotypes (frequency above 10%) was four in the control group and three in LOAD patients (Figure 2). Results of the analysis based on comparison of the haplotype frequencies showed that risk haplotypes ATAGACCG (χ^2^ = 7.313, *p* = 0.0068; OR 1.90, 95% CI 1.19–3.05) and ATAGACCA (χ^2^ = 14.589, *p* = 0.0001; OR 17.43, 95% CI 2.24–135.62) were significantly associated with LOAD. Moreover, we identified one protective haplotype GCAAGTCA (χ^2^ = 6.822, *p* = 0.009; OR 0.63, 95% CI 0.44–0.89) generated from these eight SNPs. All of the above associations remain significant after permutation test (*p* < 0.05). 

The structure of LD among eight SNPs in the *APOE-TOMM40* locus for the cases and controls is shown in Figure 1. Analysis of LD in cases and controls demonstrated that these groups were characterized by different structures of haplotype blocks at APOE-TOMM40 locus (Figure 1a,b). In controls, the first block consisted of four SNPs (rs6859, rs6857, rs157580, rs2075650) and the second included three SNPs (rs769449, rs429358, rs7412), while in the cases, the first block comprised four SNPs (rs6857, rs157580, rs2075650, rs769449) and the second block was made up of two closely located SNPs (rs429358, rs7412). Notably, this difference demonstrates the high level of LD in case group between rs429358 and rs7412 from the *APOE* gene, which are formed the APOE isoforms (ε2, ε3, ε4). The haplotype block structure for control group demonstrates the blocks that include genes: Block1-NECTIN2-TOMM40, Block2–APOE.

Although not every studied variant in genes was significantly associated with AD by univariate (case–control) analysis, there were gene–gene interactions among variants using generalized multifactor dimensionality reduction (GMDR) method. GMDR combines genotypes into “high-risk” and “low-risk” groups in order to reduce multidimensional data into only one dimension. In our study, we analyzed all possible combinations of 44 genes both in cases and in control subjects. There were three significant models as a result of the GMDR analysis: one-factor, two-factor and three-factor model (Table 5). 

**Figure 3:**
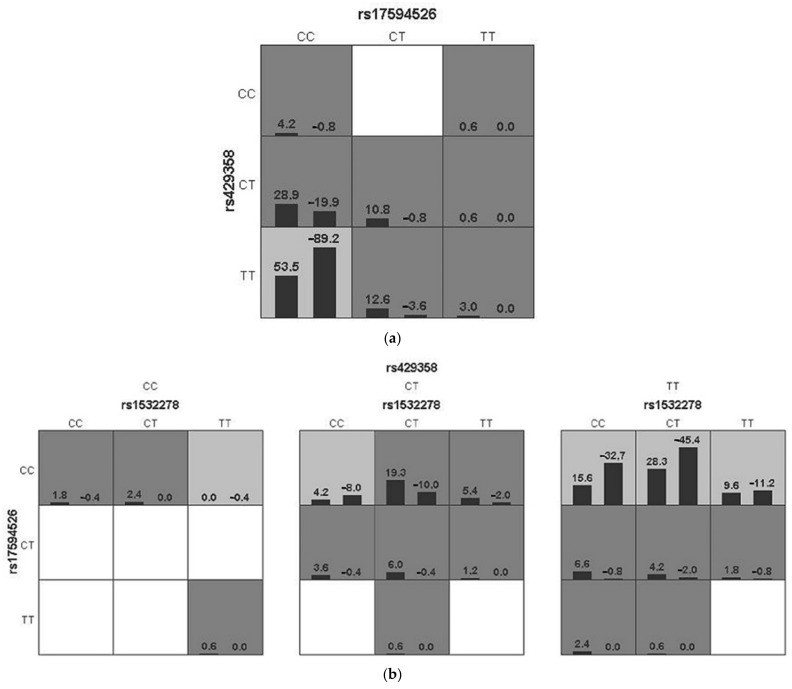
Gene–gene interactions among *TCF4* (rs17594526), *APOE* (rs429358) and *CLU* (rs1532278) loci in AD and healthy subjects: (**a**) two-factor model; (**b**) three-factor model. The score distribution of AD subjects (left black bar in boxes) and control subjects (right black bar in boxes) is shown for each genotype combination. High-risk genotype combinations are represented by dark gray shade cells, while light gray shade cells represent low-risk genotype combinations. Cells with no shading or white cells represent genotype combination for which no data is observed.

Among these results, we selected two models (a two-factor and a three-factor) as the best SNP combinations, based on the best Testing Balanced Accuracy (TBA) in data and their high cross-validation consistency (CVC) value. Figure 3 shows the score distributions in the best models. Seven genotype combinations with an increased risk of developing LOAD and only one combination of low-risk genotypes were found for the two-factor model (Figure 3a). The combination of low-risk genotypes showed statistically significant differences between the studied groups: *TCF4* rs17594526 “CC” and *APOE* rs429358 “TT” (*p* < 0.001; OR = 0.25; 95% CI: 0.14–0.45). Fourteen genotype combinations with an increased risk of developing LOAD and five combinations of low-risk genotypes were found for the three-locus model (Figure 3b). Since we did not have all genotypes for the three-locus model in GMDR analysis, we designated the data in this model as preliminary. The one-locus model was computed for *APOE* rs429358 and the empirical *p* value for prediction error using permutation testing was 0.0006, indicating the interactions among the three variants contributed to a higher risk of AD than did single variant alone (Table 5).

**Figure 4:**
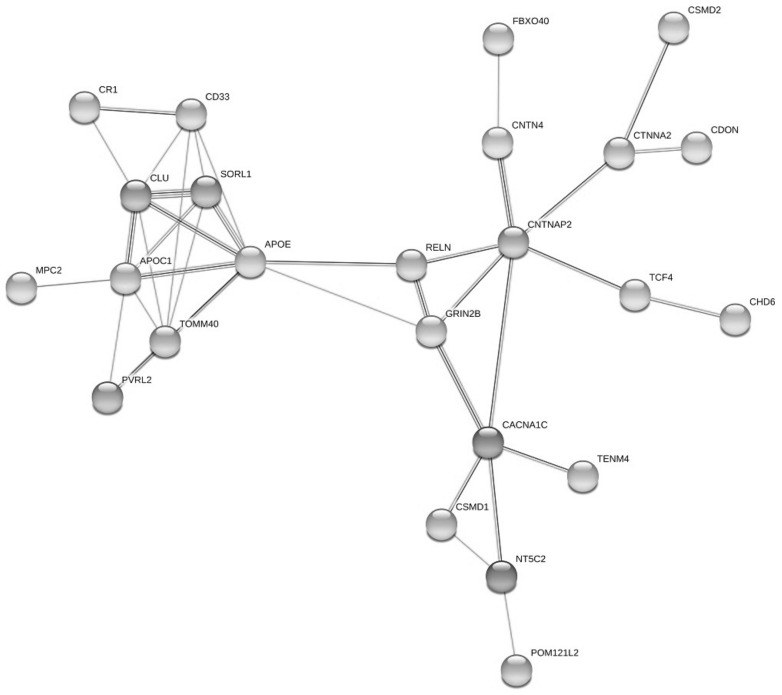
Protein–protein interaction network of studied genes. The nodes and edges represent the proteins (genes) and their interactions, respectively. The PPI network contained 24 nodes and 39 edges. The STRING indicated that the proteins formed three subnetworks of interconnected gene products. Each subnetwork had a central protein: APOE, CACNA1C, CTNNA2. All these three subsystems are connected together by RELN as a bridge.

In the present study, we conducted a protein–protein network interaction (PPI) search with proteins of AD, schizophrenia and cognitive performance collected via the GWAS database using STRING (v11.0). This exploratory bioinformatic analysis demonstrated a potential network of interaction between proteins connected to AD, schizophrenia and cognitive performance. PPI network analysis of 41 studied genes showed statistically significant connectivity among proteins. Interaction enrichment had *p*-value < 1.0 × 10^−16^ for our gene set. This means that such enrichment demonstrates that the proteins are at least partly biologically connected, as a group. The PPI network contained 24 nodes and 39 edges (Figure 4). The STRING indicated that the proteins formed three subnetworks of interconnected gene products (Figure 4). Each subnetwork had a central protein. The first subnetwork consisted of 10 proteins with APOE in the center: CD33, CR1, CLU, RELN, SORL1, NECTIN2 (PRVL2), TOMM40, APOC, MPC2. The second subnetwork included 6 proteins with CACNA1C as the center component: GRIN2B, TENM4, CSMD1, NT5C2, POM121L2. In addition, the third protein subgroup had 8 nodes with CTNNA2 in the center part: FBXO40, CNTN4, CTNNA2, CDON, CAMD2, TCF4, CHD6. Proteins with a large number of interactions were identified (with edges from 8 to 5). The eight genes with strong connections (“multiple interactions”) were *APOE, SORL1, APOC1, CD33, CLU, TOMM40, CNTNAP2* and *CACNA1C* (Figure 4). The largest number of connections was established for the APOE protein (8). This large number of interactions is not surprising, given the high known effect of the *APOE* gene for phenotypic modifications for several medical traits [23,24,25]. In addition to proteins with a large number of interactions, there are other proteins that can connect or act as bridges between subnetworks. The four “bridge” genes were *APOE*, *RELN*, *CNTNAP2* and *GRIN2B*. It should be noted that *APOE* and *CNTNAP2* represent both types of genes (“multiple interactions” and “bridge”).

To evaluate molecular function, cellular component, and biological process of our gene set we used bioinformatics resource Gene Ontology (GO, http://geneontology.org, accessed on 5 August 2021) databases via PANTHER (The Protein ANalysis THrough Evolutionary Relationships, http://www.pantherdb.org, accessed on 5 August 2021). This system includes correction for multiple testing for all GO categories, thus providing statistically significant evidence for their involvement in disease susceptibility. The studied gene set was enriched in three categories: molecular function (cell adhesion molecule binding), cellular component (neuron part), and biological process (nervous system development). Pathway enrichment analysis of genes using STRING is presented in Table 6.

## 4. Discussion

AD is the most common form of age-related neurodegenerative disease. To study such complex diseases, it is necessary to apply various types of approaches. In this study, we performed several different analyses to excavate loci involved in development of the late onset AD in the Russian population. First of all, we focused on the potential associations of SNPs previously identified in GWAS. Since not only AD is characterized by a decrease in cognitive function, we also included GWAS loci for other reported traits in our analysis, such as schizophrenia, unipolar depression, bipolar disorder, attention deficit hyperactivity disorder, autism spectrum disorder and others. 

Similar study designs have been reported in other papers for various disorders: AD and Parkinson’s disease, AD and ischemic stroke, schizophrenia and bipolar disorder, and others [26,27]. A recent study demonstrated an unbiased link between polygenic risk for schizophrenia and a lower risk of psychosis in AD [28]. The most significant associations with LOAD were observed in the APOE region on chromosome 19 (*p* < 5 × 10^−8^). Outside the APOE region, the most significant association was found in *CR1* gene on chromosome 1 (chr1:207611623, rs3818361, G/A, *p* = 6 × 10^−3^). This gene is one of the ten highest risk factors for developing AD. The *CR1* gene encodes the complement receptor 1 (CR1), which is one of the regulators of complement activity. The *CR1* might prevent excessive complement activation. The presence of *CR1* on erythrocytes is noted as an important component in protecting tissues against immune-complex deposition and following disease, such as AD [29]. 

After correction for multiple testing (the Benjamini and Hochberg false discovery rate method), only the *APOE* region genetic markers were remained statistically significant. Interestingly, according to our data, the G allele frequency (28%) of rs2075650 at *TOMM40* gene in our Russian sample sets is significantly higher than in other populations not only of European descent (13–16%), but also of the Asian population (10–12%). Salakhov et al. [30] found that *TOMM40* gene rs2075650 was associated with low-density lipoprotein cholesterol levels in healthy men group of the Russian population (Kemerovo, Russia), and G allele frequency in the total group was 21%. It should be noted that, most of the genetic variants contributing to risk of developing AD are located in non-coding regions and, presumably, they may influence as a controller of the functional activity of closely located genes that have an activating effect on transcription [31]. We can suggest that there may be some additional genetic signal in this locus that is impact to risk AD. The study results supplement the previous data on the AD risk development genetic markers in the Russian population [32].

Within the chromosomal region 19q12-q13.33, there is an accumulation of SNPs that are probably critical for beginning and development of AD. A huge number of publications devoted to the role of this region in the progress of AD. In addition, although *APOE* was identified as a susceptibility factor for LOAD over 25 years ago [33], it is still not clear how the ε4 allele contributes to disease risk. Some observations indicate an independent effect of SNPs of the NECTIN2-TOMM40-APOE locus [34], while other studies showed that there are general effects of several SNPs [35]. Our data demonstrated that in both the cases and the controls rs157580 of *TOMM40* gene is in high pairwise LD with the SNPs rs2075650 and rs769449 (D’ = 100). However, LOAD-affected individuals are characterized by higher pairwise LD between neighboring SNPs compared with unaffected individuals (Figure 1). Notably, the haplotype block structure for cases demonstrates that Block2 included rs429358 and rs7412 from the *APOE* gene, which are formed the *APOE* isoforms (*ε2, ε3, ε4*). Our study showed that the haplotypes that include the *ε3* allele of *APOE* and the A allele of *TOMM40* gene rs2075650 are associated with reduced risk for LOAD compared with haplotypes that include the *ε4* allele of *APOE* and the G allele of rs2075650. In a recently published study on heritability of extreme longevity in human populations, it was found that the *ε4* allele of *APOE* is associated with substantial reduction in the chance for extreme longevity [36].

Haplotypes that showed an association with a decrease in extreme longevity in Sebastiani et al. in our study demonstrated a significant association with an increased risk of developing LOAD. The possible combinations of loci that may reduce the risk of LOAD development were obtained by researching gene–gene interactions with GMDR. Approximately two-fold reduced risk for LOAD was observed for the combination of *CLU* rs1532278 “CC”, *TCF4* rs17594526 “CC”, *APOE* rs429358 “TT” genotypes in the three-locus model. In the model combining *TCF4* and *APOE*, genotype combination CC/TT could similarly be considered to protective to LOAD. Notably, our data presented intergenic interaction between the three loci (CLU, TCF4, APOE), demonstrating no significant associations in our replicative analysis. Transcription factor 4 gene (TCF4) plays an important role in nervous system development. TCF4, a basic helix–loop–helix transcription factor, is broadly expressed and is critical for normal brain development. The gene has been identified as the cause of Pitt-Hopkins syndrome (PTHS), and it has been implicated in various other neuropsychiatric diseases, including schizophrenia, autism and depression [37,38,39].

The STRING database aims to combine all known and predicted associations between proteins, including both functional associations and physical interactions. In the present study, we used this exploratory bioinformatic analysis to predict a possible PPI network between neurological and mental diseases with using GWAS database. The network shows possible PPIs between associated genes of AD, schizophrenia and cognitive performance. A protein–protein interaction network with three subnetworks was formed for 24 proteins. A more detailed analysis of subnetworks detected the traits of protein grouping for each of them. The first group (with the APOE) is characterized by the maximum number of edges (21). Proteins of this subnetwork are related by common biological processes, such as regulation (positive and negative) of various processes and molecule transport, as well as such common molecular function as binding. There were no significant GO associations for the other protein subgroup (the CACNA1C as a center node), but these proteins were connected through publication studies with psychiatric diseases, such as schizophrenia, bipolar disorder, depression, and others [40,41,42]. The grouping trait in the third subnetwork (with the CNTNAP2 in the center) is the biological processes related with the neuron development and differentiation, as well as with the regulation of these processes. In addition, all these three subsystems are connected together by the Reelin as a bridge. Reelin is a large signaling protein that is engaged in a cascade of cytoplasmic events that control the migration of neurons during brain development and it is essential for the correct development and plasticity of the cerebral cortex and regulate synaptic plasticity, neurotransmission and memory in the adult brain. Presumably, Reelin plays a significant role both in AD and in psychiatric disorders [43,44,45].

The PPI networks have limited clinical predictive value, but discover hypothetical new paths of interaction. The forecasted PPI network needs careful interpretation and warrants preclinical and clinical validations. Nevertheless, this network gives new facts that will be helpful to new studies of general molecular background between neurological and mental diseases.

## 5. Conclusions

In summary, genes and proteins affecting cognitive function are of special interest in the search of genetic composition of AD. The vast majority of cases of AD are viewed as sporadic results from the complex interaction of genetic risk factors and unknown environmental conditions. The present study confirmed the importance of the APOE-TOMM40 locus as the main risk locus of development and progress of LOAD. Association analysis and bioinformatics approaches detected interactions both at the association level of single SNPs and at the level of genes and proteins. Strong evidence for association with LOAD was seen for the APOE-4 polymorphism, as well as for rs429358, rs769449 and rs2075650, using various approaches: replicative association analysis, LD analysis, GMDR analysis and protein–protein interaction analysis.

Currently, priority approaches are aiming not only to search for genes, but also to establish their functional effect. This provides the possibility of using the progress made in identifying the biological effects of risk alleles to recognize new protective mechanisms against more rapid cognitive decline in AD. Identifying biological pathways that increase risk may be useful in the future for the treatment of cognitive and behavioral disorders. Nevertheless, the current findings are a step forward in in this direction. We believe that the identification of additional risk loci would be useful from the development of predictive approaches of AD.

## Figures and Tables

**Table 1:** Demographic parameters of the studied groups.

Parameter	Patients with LOAD, *n* = 185	Control Group, *n* = 287
Gender: Women	120 (64.86%)	200 (69.69%)
Gender: Men	65 (35.14%)	87 (30.31%)
Mean age	72.15 ± 7.87	71.8 ± 5.70
Race	Caucasoid	Caucasoid
Population	Russians	Russians

**Table 2:** Frequencies of the 58 SNPs analyzed for LOAD.

Gene.	SNP ID	Allele ^a^	Functional Consequence (NCBI)	Position (GRCh38)	Minor Allele Frequency		MAF
					Cases	Controls	HapMap
*CHD6*	rs1010304	G/A	intron variant	20:41473007	0.06	0.05	0.06
*NCAPD3*	rs1031381	T/C	intron variant	11:134218788	0.40	0.43	0.42
*MPC2*	rs10489202	T/G	intron variant	1:167933841	0.21	0.20	0.22
*CSMD1*	rs10503253	A/C	intron variant	8:4323322	0.23	0.27	0.26
*CCDC60*	rs11064768	G/A	intron variant	12:119380704	0.08	0.07	0.07
*NT5C2*	rs11191580	C/T	intron variant	10:103146454	0.09	0.09	0.11
*LOC105378889-PRMT6*	rs12125971	T/C	intergenic variant	1:106921021	0.08	0.08	0.07
*LOC101928778-LOC105371627*	rs12140439	A/C	intergenic variant	1:177753772	0.29	0.30	0.34
*TENM4*	rs12290811	A/T	intron variant	11:79372576	0.12	0.12	0.14
*LUZP2*	rs12361953	G/T	intron variant	11:24589584	0.14	0.14	0.15
*CADM2*	rs12494658	C/T	intron variant	3:85825326	0.24	0.30	0.28
*SNX29*	rs12922317	G/A	intron variant	16:11983775	0.40	0.35	0.35
*LOC105373605*	rs12989701	A/C	intron variant	2:127130409	0.10	0.15	0.15
*CTNNA2*	rs13034462	G/T	intron variant	2:79892368	0.03	0.04	0.05
*BRD1*	rs138880	C/A	intron variant	22:49824963	0.21	0.21	0.23
*DCHS2*	rs1466662	A/T	intron variant	4:154426241	0.35	0.35	0.69
*CLU*	rs1532278	T/C	intron variant	8:27608798	0.44	0.39	0.38
*TOMM40*	rs157580	G/A	intron variant	19:44892009	0.34	0.36	0.35
*LOC730100*	rs1606974	A/G	intron variant	2:51646461	0.08	0.06	0.07
*NKAPL*	rs1635	T/G	missense variant	6:28259826	0.07	0.06	0.05
*LSM1*	rs16887244	G/A	intron variant	8:38173827	0.23	0.21	0.21
*POM121L2*	rs16897515	A/C	missense variant	6:27310241	0.09	0.11	0.12
*CNTN4*	rs17194490	T/G	intron variant	3:2506102	0.18	0.15	0.14
*ARHGAP31*	rs17203055	G/A	intron variant	3:119365484	0.11	0.10	0.12
*TCF4*	rs17512836	C/T	intron variant	18:55527730	0.008	0.02	0.05
*CADM2*	rs17518584	C/T	intron variant	3:85555773	0.34	0.32	0.30
*TCF4*	rs17594526	T/C	intron variant	18:55391007	0.01	0.02	0.05
*GPR89P-* *TRV-AAC1-5*	rs17693963	C/A	intergenic variant	6:27742386	0.06	0.06	0.08
*TOMM40*	rs2075650	G/A	intron variant	19:44892362	0.26	0.28	0.16
*CLU*	rs2279590	T/C	intron variant	8:27598736	0.44	0.40	0.39
*ZNF365*	rs2393895	C/A	intron variant	10:62579087	0.23	0.23	0.20
*CSMD1*	rs2616984	G/A	intron variant	8:4625619	0.33	0.31	0.29
*DNAH11*	rs368331	G/A	intron variant	7:21703356	0.07	0.06	0.06
*FBXO40*	rs3772130	G/A	intron variant	3:121625293	0.20	0.25	0.25
*STK24*	rs3783006	C/G	intron variant	13:98458955	0.46	0.48	0.47
*CR1*	rs3818361	A/G	intron variant	1:207611623	0.33	0.25	0.25
*CD33*	rs3826656	G/A	intron variant	19:51223357	0.24	0.24	0.23
*APOE*	rs429358	C/T	missense variant	19:44908684	0.22	0.10	0.13
*ACSM1*	rs433598	T/C	intron variant	16:20668884	0.35	0.37	0.34
*APOC1*	rs4420638	G/A	500B Downstream Variant	19:44919689	0.18	0.14	0.18
*CNTN4*	rs4629318	A/G	intron variant	3:2851590	0.15	0.15	0.14
*CDON*	rs472926	G/A	intron variant	11:126035363	0.16	0.17	0.17
*CACNA1C*	rs4765905	C/G	intron variant	12:2240418	0.32	0.37	0.37
*TENM4*	rs530965	T/C	intron variant	11:79354056	0.49	0.47	0.49
*CSMD2*	rs544991	T/C	intron variant	1:33723829	0.31	0.27	0.31
*PICALM-* *RNU6-560P*	rs561655	G/A	intergenic variant	11:86089237	0.30	0.35	0.33
*CHD6*	rs6129846	T/C	intron variant	20:41478674	0.06	0.05	0.06
*NRXN3*	rs6574433	G/A	intron variant	14:78319816	0.45	0.42	0.42
*CR1*	rs6656401	A/G	intron variant	1:207518704	0.31	0.23	0.24
*NECTIN2*	rs6857	T/C	3 Prime UTR Variant	19:44888997	0.28	0.19	0.17
*NECTIN2*	rs6859	A/G	intron variant	19:44878777	0.50	0.42	0.44
*LOC105375630*	rs7004633	G/A	intron variant	8:88748082	0.21	0.19	0.20
*RELN*	rs7341475	A/G	intron variant	7:103764368	0.16	0.19	0.16
*LOC105373605*	rs7561528	A/G	intron variant	2:127132061	0.31	0.31	0.34
*APOE*	rs769449	A/G	intron variant	19:44906745	0.17	0.08	0.10
*KLHL1*	rs7984606	C/A	intron variant	13:69881529	0.003	0.004	0.05
*NKAIN2*	rs9491140	T/C	intron variant	6:124370091	0.32	0.32	0.31
*APOE*	rs7412	T/C	missense variant	19:44908822	0.08	0.07	0.07

^a^ Minor allele/major allele.

**Table 3:** Association of statistically significant genetic markers with LOAD in the Russian population.

Gene	Variant	Minor Allele	OR (95% CI)	Major Allele	OR (95% CI)	*p* Value	*p* Value Corrected
*APOE4* * (ε3/ε4)	rs429358 rs7412	ε4	2.88 (1.95–4.24)	ε3	0.35 (0.24–0.51)	5 × 10^−8^	8.62 × 10^−4^
*APOE* *	rs769449	A	2.44 (1.62–3.66)	G	0.41 (0.27–0.62)	1 × 10^−5^	2.5 × 10^−3^
*APOE* *	rs429358	C	2.53 (1.75–3.67)	T	0.39 (0.27–0.57)	5 × 10^−7^	1.7 × 10^−3^
*TOMM40* *	rs2075650	G	1.67 (1.21–2.29)	A	0.60 (0.44–0.82)	2 × 10^−3^	3.4 × 10^−3^
*NECTIN2* *	rs6857	T	1.65 (1.21–2.26)	C	0.61 (0.44–0.83)	2 × 10^−3^	4.3 × 10^−3^
*CR1*	rs3818361	A	1.5 (1.12–2.00)	G	0.67 (0.5–0.89)	6 × 10^−3^	5.2 × 10^−3^
*CR1*	rs6656401	A	1.46 (1.09–1.96)	G	0.68 (0.51–0.92)	0.01	6 × 10^−3^
*NECTIN2*	rs6859	A	1.34 (1.03–1.74)	G	0.75 (0.57–0.97)	0.03	6.8 × 10^−3^
*FBXO40*	rs3772130	G	0.72 (0.52–0.99)	A	1.39 (1.01–1.91)	0.04	7.7 × 10^−3^

*OR* odds ratio, *CI* confidence interval, *p* values corrected based on Benjamini and Hochberg method, * Statistically significant results after multiple comparison correction.

**Table 4:** Identical haplotypes identified in both groups.

N	rs, Gene								Haplotype Frequencies (Cases/Controls) %
	rs6859 *NECTIN2*	rs6857 *NECTIN2*	rs157580 *TOMM40*	rs2075650 *TOMM40*	rs769449 *APOE*	rs429358 *APOE*	rs7412 *APOE*	rs4420638 *APOC1*	
1	G	C	G	A	G	T	C	A	21.4/26.3
2	A	C	A	A	G	T	C	A	15.7/16.4
3	G	C	A	A	G	T	C	A	14.0/20.9
4	A	T	A	G	A	C	C	G	11.0/6.4
5	A	T	A	G	G	T	C	A	9.1/9.9
6	A	C	G	A	G	T	C	A	7.1/5.5
7	G	C	G	A	G	T	T	A	5.0/3.1
8	G	C	A	A	G	T	T	A	2.8/3.0
9	A	C	A	A	G	T	C	G	2.3/2.7
10	G	T	A	G	A	C	C	G	1.4/1.0
11	G	T	A	A	G	C	C	G	1.1/0.6
12	G	C	A	A	A	C	C	G	0.7/0.6
13	A	T	A	A	G	C	C	G	0.7/0.8
14	G	C	A	A	G	T	C	G	0.5/1.0

**Table 5:** Gene–gene interaction models by GMDR analysis.

Best Interaction Model	TBA ^#^	CVC	*p* Value	OR ^#^ (95% CI) ^#^
APOE	0.59	6/10	0.0006	2.81 (1.54–5.13)
*TCF4, APOE*	0.66	10/10	<0.0001	4.04 (2.27–7.17)
*CLU, TCF4, APOE*	0.66	10/10	<0.0001	5.71 (3.03–10.78)

CVC—cross validation consistency, TBA—testing balanced accuracy, ^#^ Values rounded up to 2 decimal places.

**Table 6:** Significantly enriched pathways of studied genes.

Pathway ID	Pathway Description	Observed Gene Count	False Discovery Rate	Matching Proteins in Your Network (Labels)
GO:0022008	neurogenesis	15	0.00028	APOE,CDON,CLU,CNTN4,CNTNAP2,CTNNA2,KLHL1,LSM1,NRXN3,RELN,SORL1,STK24,TCF4,TENM4,ZNF365
GO:0007155	cell adhesion	11	0.00036	CADM2,CD33,CDON,CNTN4,CNTNAP2,CTNNA2,DCHS2,NRXN3,PVRL2,RELN,TENM4
GO:0007399	nervous system development	17	0.00036	APOE,CDON,CLU,CNTN4,CNTNAP2,CTNNA2,DNAH11,GRIN2B,KLHL1,LSM1,NRXN3,RELN,SORL1,STK24,TCF4,TENM4,ZNF365
GO:0048699	generation of neurons	14	0.00036	APOE,CDON,CNTN4,CNTNAP2,CTNNA2,KLHL1,LSM1,NRXN3,RELN,SORL1,STK24,TCF4,TENM4,ZNF365
GO:0032989	cellular component morphogenesis	10	0.00044	CLU,CNTN4,CNTNAP2,CTNNA2,NRXN3,PVRL2,RELN,STK24,TENM4,ZNF365
GO:0050767	regulation of neurogenesis	10	0.00044	APOE,CDON,CNTN4,LSM1,RELN,SORL1,STK24,TCF4,TENM4,ZNF365
GO:0048666	neuron development	10	0.00045	APOE,CNTN4,CNTNAP2,CTNNA2,KLHL1,NRXN3,RELN,STK24,TENM4,ZNF365
GO:0031175	neuron projection development	9	0.00058	APOE,CNTN4,CNTNAP2,CTNNA2,KLHL1,NRXN3,RELN,STK24,ZNF365
GO:0000902	cell morphogenesis	9	0.00061	CLU,CNTN4,CNTNAP2,CTNNA2,NRXN3,RELN,STK24,TENM4,ZNF365
GO:0032990	cell part morphogenesis	8	0.00064	CNTN4,CNTNAP2,CTNNA2,NRXN3,PVRL2,RELN,STK24,ZNF365
GO:0007417	central nervous system development	10	0.00084	CDON,CLU,CNTN4,CNTNAP2,CTNNA2,GRIN2B,KLHL1,RELN,TENM4,ZNF365
GO:0007611	learning or memory	6	0.00089	APOE,CNTNAP2,DNAH11,GRIN2B,NRXN3,RELN
